# Carbonic Anhydrase Inhibitors and Epilepsy: State of the Art and Future Perspectives

**DOI:** 10.3390/molecules26216380

**Published:** 2021-10-22

**Authors:** Lidia Ciccone, Chiara Cerri, Susanna Nencetti, Elisabetta Orlandini

**Affiliations:** 1 Department of Pharmacy, University of Pisa, via Bonanno 6, 56126 Pisa, Italy; lidia.ciccone@unipi.it (L.C.); chiara.cerri@unipi.it (C.C.); 2 Department of Earth Sciences, University of Pisa, via Santa Maria 53, 56126 Pisa, Italy; 3 Research Center “E. Piaggio”, University of Pisa, 56122 Pisa, Italy

**Keywords:** carbonic anhydrases (CAs), CA inhibitors (CAIs), epilepsy, CA II, CA VII, CA XIV

## Abstract

Carbonic anhydrases (CAs) are a group of ubiquitously expressed metalloenzymes that catalyze the reversible hydration/dehydration of CO_2_/HCO_3_. Thus, they are involved in those physiological and pathological processes in which cellular pH buffering plays a relevant role. The inhibition of CAs has pharmacologic applications for several diseases. In addition to the well-known employment of CA inhibitors (CAIs) as diuretics and antiglaucoma drugs, it has recently been demonstrated that CAIs could be considered as valid therapeutic agents against obesity, cancer, kidney dysfunction, migraine, Alzheimer’s disease and epilepsy. Epilepsy is a chronic brain disorder that dramatically affects people of all ages. It is characterized by spontaneous recurrent seizures that are related to a rapid change in ionic composition, including an increase in intracellular potassium concentration and pH shifts. It has been reported that CAs II, VII and XIV are implicated in epilepsy. In this context, selective CAIs towards the mentioned isoforms (CAs II, VII and XIV) have been proposed and actually exploited as anticonvulsants agents in the treatment of epilepsy. Here, we describe the research achievements published on CAIs, focusing on those clinically used as anticonvulsants. In particular, we examine the new CAIs currently under development that might represent novel therapeutic options for the treatment of epilepsy.

## 1. Introduction

According to the International League Against Epilepsy (ILAE), epilepsy is a chronic brain disorder operationally defined by the occurrence of two unprovoked seizures more than 24 h apart, or one unprovoked seizure when the risk for another is known to be high (>60%) [1]. Seizures can manifest in a variety of different clinical presentations with motor, sensory, autonomic or psychic origin [2]. Seizure episodes are a result of abnormal excessive or synchronous neural activity in the brain. Seizures are classified into focal and generalized types. Focal seizures are localized in a specific cerebral area. Thus, the behavioral outcome depends on the brain regions where synchronous firing of a neuronal cell group occurs. Generalized seizures, spreading through thalamocortical connections, involve both cerebral hemispheres. Considering the specific symptoms and etiology, subclassifications of epileptic seizures are also reported [3].

Epilepsy affects 50 to 70 million people of all ages worldwide. The prevalence and incidence of epilepsy tend to peak in childhood (where causes generally are genetic and where remission, spontaneous or after adequate treatment, could occur later in life) and, even more markedly, in the elderly (where epilepsy is a consequence of those brain insults that are frequent at this age-group: stroke, neurodegenerative disease, brain tumors).

People with epilepsy and their families can still be targets of stigma and prejudice today with consequent social discriminations. This is particularly evident in low- and middle-income countries where 75% of the people affected do not receive the treatment they need because of economic as well as cultural circumstances [4].

The high impact of the disease on global health has provoked immense efforts from the scientific community to shed light on the complex mechanisms underlying seizure generation and to develop therapeutic strategies to pharmacologically treat epilepsy. However, antiepileptic drugs (AED) currently available and employed in clinical practice can treat only some subtypes of epilepsy and, often, pharmacological treatment may not be resolute [5]. For this reason, there is an urgent need to identify new molecular targets in order to expand the therapeutic options to treat and to defeat this dramatic pathology [4,6,7].

In the last years, research in this field has turned the spotlight on the role of carbonic anhydrases (CA) in epilepsy and it has emerged as an attractive target for designing new anticonvulsant drugs [8,9,10].

In humans, sixteen isoforms of α-CAs exist that have different catalytic activities and various subcellular and tissue distribution. In particular, hCA I–III, hCA VII and hCA XIII are cylosolic isoforms, hCA IV, hCA IX, hCA XII, hCA XIV and hCA XV are membrane bound, hCA VA and hCA VB are mitochondrial isoforms and hCAVI is a secreted isoform [8].

**Figure 1:**
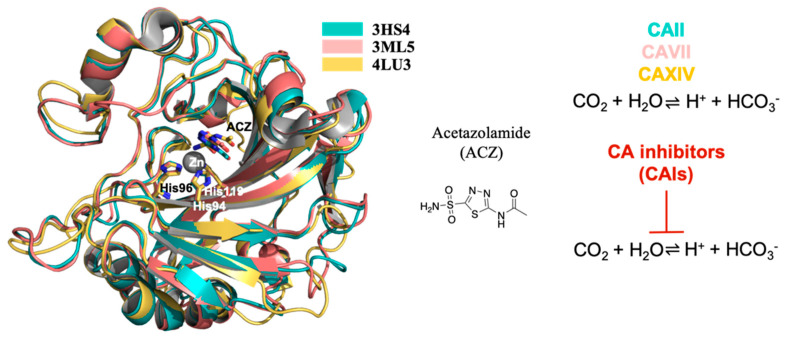
Superposition of human CAII, VII and XIV in complex with acetazolamide (ACZ). In the middle chemical structure of ACZ, on the right CAs activity and CAIs action. All the structural figures have been made with PyMol [12] modifying the scripts previously used [13,14] and the images were assembled using GNU Image Manipulation Program (GIMP) [15].

The active site of α-CAs is characterized by a zinc ion (Zn^2+^) that is crucial for the catalytic reaction [11]. These metalloenzymes catalyze the reversible hydration/dehydration of CO_2_/HCO_3_ (Figure 1), regulate CO_2_ tissue concentration as well as cellular pH buffering.

More recentlySince it has been clearly demonstrated that low CO_2_ levels and alkalosis increase neural excitability, sustaining seizure generation [16,17,18], CAs have been considered as important players in the pathogenesis of epilepsy.

In addition, it is known that epileptiform events can be driven by excitatory GABA. GABA is the main inhibitory neurotransmitter in the brain, but under certain conditions and states, including early developmental stages and epilepsy, GABA may exert depolarizing actions [19,20]. It has been reported that GABAergic excitation is related to CA activity, especially CA II and CA VII [21].

In this scenario, the inhibition of some isoforms of CA enzymes has been proposed and exploited as a therapeutic approach to modulate and control abnormal epileptic activity. CA inhibitors (CAI) show a good anticonvulsant profile and four molecules are actually clinically used in epilepsy therapy: acetazolamide (ACZ), see Figure 1, zonisamide (ZNS), methazolamide (MZA) and topiramate (TPM) [9]. However, the exact mechanism by which they act is not completely understood yet.

Here, we review the state of the art relative to the development of selective CA inhibitors (CAI) as anticonvulsants. In particular, we describe the known CAI actually used in clinical practice and the progress made in the design of new selective inhibitors of those CA particularly critical for epilepsy (i.e., isoforms II, VII and XIV) [22], Figure 1.

## 2. Structure of Carbonic Anhydrase (CA): CAII, CAVII and XIV

The catalytic mechanism is more or less the same in all α-Cas, and it has been largely studied using CA II as a model. The catalysis happens in two steps following the reactions reported below

E-Zn^2+^-OH^−^ + CO_2_ ⇋ E-Zn(OH^−^)CO_2_^−^ ⇋ E-Zn^2+^-HCO_3_^−^⇋ E-Zn^2+^-H_2_O + HCO_3_^−^ (1)

E-Zn^2+^-H_2_O ⇋ E-Zn^2+^-OH^−^ + H^+^ (2)

The CA enzyme is active when it is in its basic form, or rather, the hydroxide group is bound to Zn^2+^ (E-Zn^2+^-OH^−^) (1) [23,24]. The complex E-Zn^2+^-OH^−^ is responsible for the nucleophile attack on the CO_2_ molecule, located in the hydrophobic region beside the zinc ion, leading to the formation of HCO_3_^−^. Then, the bicarbonate ion is released in solution and replaced by a water molecule (E-Zn^2+^-H_2_O) generating the inactive form. To activate the enzyme again, a new transfer of protons from the E-Zn^2+^-H_2_O complex is needed (2). The latter is the rate-limiting reaction step of the catalytic turnover.

Hence, in all α-CAs, the active conformation is conducted by a metal hydroxide species L_3_-M^2+^-OH^−^ (L_3_ are the amino acids that coordinate the metal ion M^2+^) that makes the nucleophile attack with a CO_2_ molecule located in the adjacent hydrophobic pocket [25,26].

**Figure 2:**
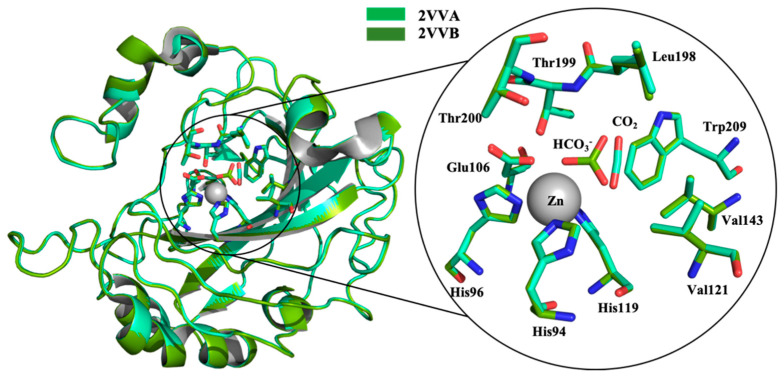
Superposition of CA II crystal structure in complex with CO_2_ (pdb 2VVA) and HCO_3_^−^ (pdb 2VVB); limegreen and splitpea, respectively. In the circle, focus of the catalytic domain.

Recently, crystallographic studies have clarified how the two key substrates, CO_2_ and HCO_3_^−^, bind the CA’s active site. In particular, the crystal structure of α-CA II (pdb 2VVA) displays that CO_2_ is located in the hydrophobic pocket (around Val121, Val143, Trp209 and Leu198) and it does not directly bind the Zn^2+^ ion, while the HCO_3_^−^ specie coordinates metal ion (pdb 2VVB) [27] (Figure 2). 

The sequence of the hCAs’ catalytic domains is highly maintained among several isoenzymes, and this reflects in a high degree of three-dimensional structural similarity (CA I, II, III, IV, V, XII, XIII and XIV) [28,29]. 

**Figure 3:**
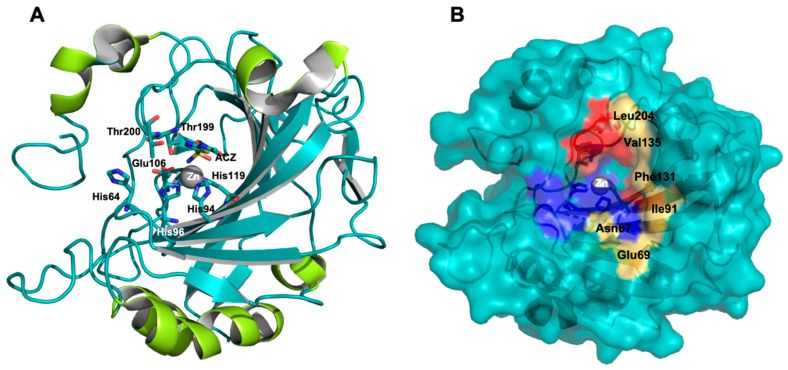
Graphical representation of CA II (pdb id 3HS4). (**A**) CA II in complex with ACZ, α-helices are colored in lemon. (**B**) Protein surface of CA II, hydrophobic residues of catalytic site are colored in red, hydrophilic residues are colored in blue while the unique amino acid residues of CA II are highlighted yellow–orange and labeled.

CA II is a polypeptides chain of 259 amino acids (29.3 KDa) characterized by central 10-stranded twisted ß-sheet structures and six right-handed α-helices on a molecular surface. The ß-strands are all parallel except for two pairs that are antiparallel; moreover, there are some ß-turns along the large ß-structure [28,29] (Figure 3A).

CA II, as well as all other CAs, has the canonical three-dimensional ellipsoidal fold [30] (Figure 3A). The active site is characterized by a Zn^2+^ ion, fundamental for catalysis, placed at the bottom of the canonic cleft that is composed of a hydrophobic and a hydrophilic pocket (Figure 3B) [25]. 

The Zn^2+^ ion is stabilized by the interaction with the imidazole side chains of three conserved His residues (His94, His96 and His119) and with one water molecule (or hydroxide ion (OH^−^) according to pH values). In detail, the H_2_O or OH^−^ takes part in a hydrogen bond network that requires another water molecule “deep water” and the OH group of Thr199, a residue conserved in all hCAs [31]. Moreover, the OH moiety of Thr199 is involved in a hydrogen bond with COOH group of Glu106, a residue always present in α-CAs. The H_2_O or (OH^−^) is the molecule replaced by the classical Zn^2+^ chelator inhibitors (Figure 3A) [32,33]. 

**Figure 4:**
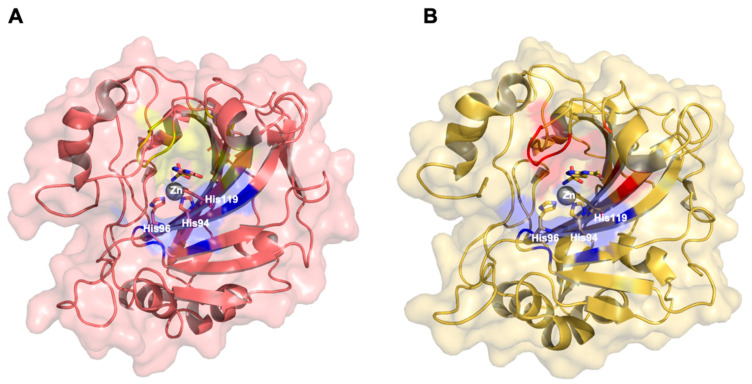
Crystal structure of CA VII and CA XIV in complex with ACZ. (**A**) The ACZ chelates, together with the three conserved His, Zn^2+^ in the catalytic site of CA VII. Hydrophobic residues are colored in yellow, while hydrophilic residues are colored in blue (pdb 3ML5). (**B**) The ACZ chelates, together with the three conserved His, Zn^2+^ in the catalytic site of CA XIV. Hydrophobic residues are colored in red, while hydrophilic residues are colored in blue (pdb 4LU3).

The crystal structure of CA VII displays a similar three-dimensional arrangement of CA II. In order to prevent crystallogenesis complication, a double mutant hCA VII (Cys183 and Cys217 were muted into Ser) was produced. The crystal structure confirms that the center of the active site is constituted by 10-strands ß-sheet structured encompassed by α-helices and extra ß-sheets. Moreover, the morphology of the active site is maintained [34]. The catalytic zinc ion is located in the bottom of a conical cavity coordinated by three histidine residues, Figure 4A. Studies of molecular dynamic simulations highlighted that the active site of CA VII can be stretched with respect to the CA II catalytic cleft and, consequently, CA VII can bind bulkier inhibitors than CA II [35,36]. 

As mentioned before, CA XIV is a transmembrane protein and its catalytic site is located over the cell membrane. The hCA XIV shares 34–46% of amino acids sequence identity with the other CAs membrane isoenzymes. In 2004, the murine CA XIV crystal structure was solved by Whittington et al. [37], and several years later the hCA XIV was published by Alterio et al. [38]; both structures are very similar to each other.

The CAXIV extracellular domain, as well as the other CAs isoforms, displays an ovoidal shape. It possesses the typical fold of human α-CAs in which a 10-stranded ß-sheet builds the core of the enzyme (Figure 4B) [38].

**Figure 5:**
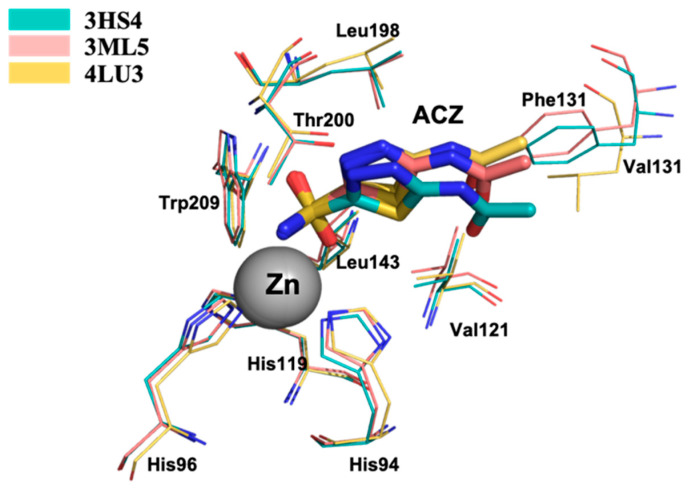
Superposition of the crystal structures CA II, CA VII and CA XIV in complex with ACZ.

The superposition among the crystal structures of CA II, VII and XIV in complex with ACZ show that ACZ binds all these isozymes in a similar mode. The sulfonamide amine N atom of ACZ binds directly to the Zn^2+^ ion, together with the side chains of His94, His96 and His119, in the catalytic cleft (Figure 5) [27,33,38]. In general, this ligand orientation, in the catalytic site, is observed for the sulfonamide CAI base inhibitors.

**Figure 6:**
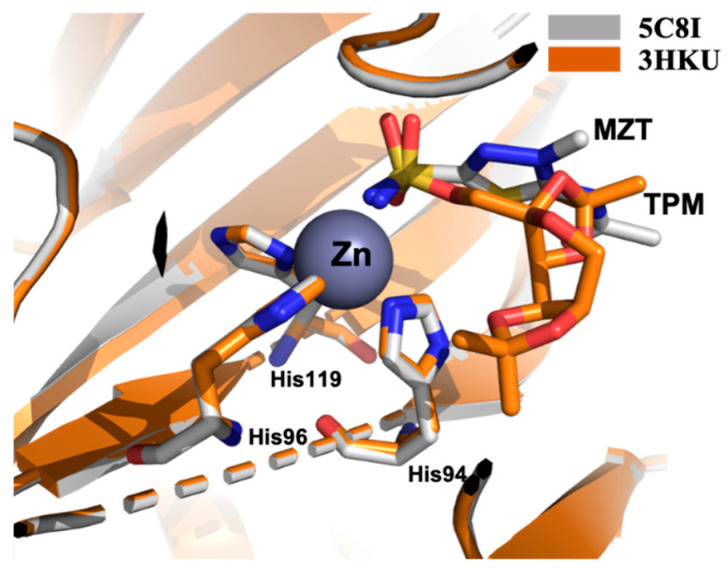
Superposition between CAII in complex with MZT (grey) and TPM (orange).

The superposition of the crystal structures of CA II in complex with MZT and TPM (pdb 5C8I and 3HKU, respectively) confirm that also for these derivatives, the sulfonamide group chelates the Zn^2+^ ion (Figure 6). This interaction directs the ligand orientation into the catalytic cleft.

## 3. CAs and Their Role in Epilepsy

CAs catalyze a crucial reaction that basically takes place in all living organisms: the reversible hydration of carbon dioxide into bicarbonate and protons CO_2_+ H_2_O ⇋ HCO_3_^−^+H^+^.

CA’s physiological function is then essential for all species. In mammals, CAs are involved in several biological processes that directly or indirectly use components of this reaction, such as respiration, pH regulation, secretion of electrolytes and HCO_3_^−^ dependent metabolic processes [39]. 

In humans and vertebrates, CAs are encoded by the α-CA gene. This class of CAs consists of 16 subtypes with different tissue and cellular locations. In the brain, several CA isoforms have been identified. CA II was the first CA isoenzyme to be associated with the brain [40]. It is expressed in neurons where it is confined to the cytoplasm [10], in oligodendrocytes [41], choroid plexus, astrocytes and in myelinated tracts [42]. The isoform CA VII has been found widely expressed in the hippocampus and cortex, exclusively at neuronal level. Other isoforms with a widespread expression throughout the brain are CA IV, with a prevalent expression in endothelial cells, the mitochondrial CA V and CA VIII, mainly found in glial cells and neurons [43,44]. Additionally, the isoforms CA III, CA X, CA IX, CA XI and CA XII have been found in cerebral tissue, although with a weak expression in normal conditions [44,45]. 

CA VIII, X and XI are three catalytically inactive carbonic anhydrase-related proteins (CARPs), which were speculated to function through interaction with other proteins [46]. Finally, CA XIV and CA XV were found located in the plasma membrane on neuronal bodies and on axons in the mouse and human brain [47,48]. 

The physiological role of cerebral CAs mainly consists in pH regulation, ion compartmentation and formation of cerebrospinal fluid.

Much of the evidence indicates that brain CAs are also involved in neuropathological processes associated with seizure generation. Epileptic conditions can increase CA levels in the brain, while their absence confers seizure resistance in animal models of epilepsy. Indeed, it has been demonstrated that CA II and CA XII levels are heightened in the epileptic brain [49] and that there is a lack of electrographic experimental induced febrile seizures in CA VII deficient mice [21]. 

**Figure 7:**
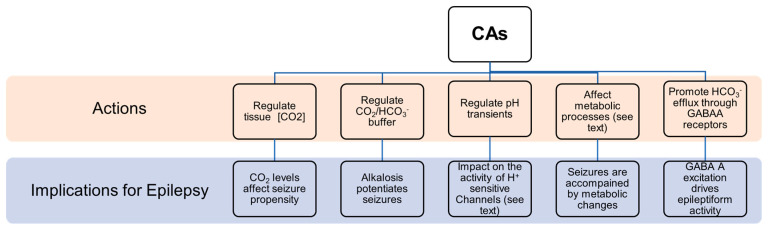
Schematic view of CAs actions and their involvement in epileptic processes.

There are multiple factors that link CAs to seizures (Figure 7):

(1) Seizures are accompanied by pronounced changes in ionic composition in brain compartments and by pH shift that, directly or indirectly, influence the concentration of the chemical species of the reaction catalyzed by CAs.
(2) CAs regulate CO_2_ tissue concentration, and it has been demonstrated that CO_2_ has a role in epilepsy. In particular, clinical evidence suggest that the enhancement of CO_2_ concentration results in better seizure control [50], while low CO_2_ levels are linked to higher seizure propensity [51]. The inhibition of CAs resulted in increased CO_2_ concentration and a positive outcome in epilepsy management [9].
(3) It has been clearly shown that alkalosis generally potentiates seizures by increasing neuronal excitability, while acidosis has an opposite effect [52]. Since their role is in the regulation of the CO_2_/ HCO_3_^−^ buffer system, CAs are crucially involved in the control of neural excitability [17]. For instance, it has been demonstrated that CA IV and CA XIV play a role in extracellular buffering in response to neural activity [53].
(4) Mitochondrial dysfunction has been identified as one potential cause of epileptic seizures [54]. There is a vicious cycle between mitochondrial dysfunction and epileptic seizures because seizures themselves can induce mitochondrial damage that consequently triggers seizures [54]. It is known that CAs are involved in mitochondria biogenesis and physiology, and in glucose and lipid metabolism in human Sertori cells [55]. In particular, CA V A and CA V B are specifically localized in mitochondria. They hydrate carbon dioxide to yield bicarbonate ions and a proton that contribute to normal mitochondria metabolism. In the nervous system, CA V is expressed in astrocytes as well as in neurons. It has been proposed that CA V in neurons could be involved in the regulation of the intra-mitochondrial Ca^2+^ levels, thus contributing to the stability of the intracellular calcium concentration preventing neuronal degeneration and cell death [43]. Another possible function of CA V is to participate in the regulation of neuronal HCO_3_^−^ homeostasis taking part in physiological neuronal function. Moreover, it has been reported that the intracellular regeneration of HCO_3_^−^ and its elimination from the extracellular environment results in a repolarization in GABA responses, suggesting that CA V might also be involved in neuronal transmission [43,56].
(5) Regulating the kinetics of pH transients [17,57,58]. CAs can influence the function of a broad array of proton-sensitive transmembrane proteins implicated in neuronal signaling such as GABAARs [57,58], N-methyl-D-aspartate (NMDA) receptors [59,60], H^+^-gated channels [61] and cation channels [62,63]. For example, the activity of excitatory receptors for glutamate, NMDA receptors, is inhibited by extracellular protons [64]. The initial seizure-associated extracellular alkaline shift, apparently influenced by CA activity [53], likely sustains NMDA receptors’ activation during seizures. Moreover, it has been shown that CA XIV, located in close vicinity to the NMDA receptor at the synapses, regulates pH transients in the perisynaptic microenvironment and their impact on NMDA receptors’ activity [60].
(6) It has been shown that glycolysis increases during seizures and that the glycolytic metabolite lactic acid can be used as an energy source [65]. A specific isoform of CAs facilitate lactate transport in astrocytes as well as in neurons [66]. In addition, CAs can intervene in lactic acid-induced acidosis, that seems to be implicated in seizure termination [65,67]. Moreover, CAs provide substrates required for the function of metabolic enzymes involved in epilepsy. For instance, a failure in pyruvate carboxylase (PC) function may lead to seizures, as demonstrated by the fact that PC deficiency is related to recurrent seizures in patients. CA V, providing HCO_3_^−^ to pyruvate carboxylase, is involved in controlling the proper functioning of this enzyme [40] and, then, its action might have implications for epilepsy.
(7) Numerous experimental and clinical studies support the notion that oxidative stress substantially contributes to the pathogenesis of epilepsy [68]. Studies showed that patients affected by epilepsy report a remarkable increase in levels of oxidative markers, such as malondialdehyde (MDA), protein carbonylation (PC) and nitric oxide (NO), when compared to a control group. An excessive production of free radicals could be implicated in neuronal hyperexcitability that triggers epileptogenesis. Moreover, it has been reported that overproduction of reactive oxygen species (ROS) provokes the progressive disruption of Ca^2+^ homeostasis essential for neuronal survival. In this context, it has been proposed that CAs, in particular CA VII might also have a role in the cell defence against oxidative damage thanks to its cysteine residues [69].
(8) GABAergic inhibition has been traditionally considered as the principal mechanism counterbalancing glutamatergic excitation and preventing epileptiform activity. Indeed, many of the currently used antiepileptic drugs act through enhancement of GABAergic signaling. However, much evidence has shown that epileptiform events can also be characterized by synchronous firing driven by excitatory GABA [70]. As during the first phases of development [19], excitatory action of GABA in epilepsy is due to (a) elevated intracellular chloride concentration as a result of chloride accumulation during hyperactivity [71]. High levels of intra-neuronal Cl^−^ leads to Cl^−^ efflux and then to depolarization in response to GABA binding to its type A receptor; (b) HCO_3_^−^ permeability of GABA-A channels [72,73] that causes HCO_3_^−^ efflux and then depolarization; (c) elevation of extracellular potassium caused by KCC2-mediated extrusion of chloride and potassium that results in membrane depolarization [74]. CAs are implicated in this abnormal epilepsy-associated GABA-A excitation. Specifically, it has been shown that they have a role in favouring the efflux of HCO_3_^−^ ions through GABA-A receptors [75,76]. CA VII, which plays an important role in the development of febrile seizures [21], has been identified as a key molecule in GABAergic excitation and it has been suggested that CA VII developmental expression governs the electrophysiological behaviour related to neural circuit plasticity and to susceptibility to epileptogenesis [77].

As demonstrated above, the evidence for the existence of a close relationship between epilepsy and CAs are numerous. However, further studies are needed to clarify and better define the exact role of CAs in epilepsy. In particular, it might be possible that some isoforms facilitate epileptic seizures while others are protective. For example, CA VII could favor seizures (see above point 8) while CA V, being involved in normal mitochondrial physiology and by providing HCO_3_^−^ to pyruvate carboxylase (point 4 and 6), might have a protective role in epilepsy. In addition, more than one isoform, independently or in concert, might contribute to seizure implementation. All of these hypotheses remain to be addressed. What is certain is that CAs represent molecular targets for epilepsy and that the interference with their activity, through specific inhibitors (CAIs), has an anticonvulsive outcome.

## 4. CA Inhibitors Clinically Employed in Epilepsy Therapy

**Figure 8:**
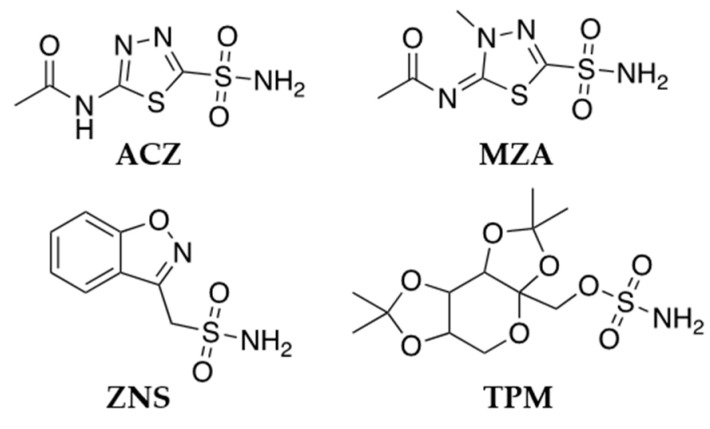
Chemical structure of the most known CA inhibitors.

Acetazolamide (ACZ), methazolamide (MZA), zonisamide (ZNS) and topiramate (TPM) are the most known CA inhibitors, belonging to the class of sulfonamides (Figure 8) that act as anticonvulsants in animal models of epilepsy as well as in epileptic patients.

ACZ, also known with the commercial name Diamox, was approved in 1953 as a diuretic. Actually, it is also indicated as an adjuvant for epilepsy treatment. In particular, it might be useful in partial, myoclonic, absence and primary generalized tonic–clonic seizures [40]. It seems to be particularly effective in the treatment of catamenial epilepsy in women [78]. However, since the possible side effects, including tinnitus, kidney stones, paresthesia, loss of appetite, alteration of taste [79] and the lack of an effective long-term therapy due to the development of tolerance in patients, ACZ is rarely used as an antiepileptic drug.

ACZ is able to inhibit various CA isoforms, including CA II, CAV, CAVII, CAXII and CA IV [40]. ACZ reduces the excitability of cortical neurons and suppresses neural discharges in the Maximal Electroshock (MES) model, an animal model of generalized tonic–clonic seizures [10,80]. Its anticonvulsant effect has been mainly attributed to its capacity to increase CO_2_ levels in the brain and to inhibit GABA-A depolarization [80,81].

The other sulfonamide methazolamide (MZA) has inhibitory proprieties similar to those of ACZ [40]. In the literature, few articles regarding the antiepileptic effect of MZA are available. Most of them date back to the 1950s–1960s [82]. MZA was studied in animals as well as in humans: this resulted in different physicochemical properties and body distribution with respect to ACZ and seemed more active than ACZ against experimental epilepsy [83]. However, very few clinical trials on the use of MZA in epilepsy have been conducted; nowadays its clinical use is mainly restricted to the treatment of glaucoma since it is able to potently reduce intraocular pressure.

Zonisamide (ZNS) was originally synthesized in Japan in 1974. Preclinical animal studies revealed its antiseizure effect on MES in rats, mice, rabbits and dogs [84]. Several controlled clinical studies conducted in the USA and Europe demonstrated ZNS’s efficacy in the treatment of partial seizures in adults. ZNS was approved by The Food and Drug Administration (FDA) in the USA in 2000 as an adjunctive therapy in the treatment of partial seizures in adults with epilepsy. Currently, it is a widely used seizure medicine, known with the common brand name “Zonegran”, particularly exploited for the treatment of Temporal Lobe Epilepsy, Focal Impaired Awareness or Complex Partial Seizures Refractory Seizures, Secondarily Generalized Seizures and Simple Partial Seizures [85].

ZNS is fairly safe, well tolerated in patients and has a better, although not yet optimal, long-term efficacy profile compared to ACZ. However, its application is often accompanied by some side effects, such as headache, nausea, somnolence, dizziness and weight loss.

ZNS anticonvulsant proprieties are attributable to various molecular mechanisms: (a) it blocks low-voltage-gated sodium channels [86,87] and T-type calcium channels [88]. Both types of voltage-gated channels are implicated in controlling neuronal firing and, then, play a role in epilepsy; (b) It upregulates GABA-mediated inhibition of seizures, while reducing excitatory glutamatergic transmission. In particular, it has been shown that ZNS increases synaptic concentrations of GABA through the regulation of glutamate and GABA transporter proteins [89] and inhibits calcium-dependent, potassium-evoked extracellular glutamate release in the hippocampus [89]. It has also been demonstrated that ZNS affects GABAergic and glutamatergic neurotransmission by acting on inositol triphosphate receptor-associated neurotransmitter release [90]; (c) it acts on dopaminergic and serotonergic transmission generating antiseizure and positive psychotropic effects [91]; (d) it has free radical scavenging activities that protect neurons from the free radical damage associated to epilepsy [92]; (e) it is a potent non-specific CA inhibitor. As we reported above, it has been demonstrated that CAs are implicated in seizure generation, so, presumably, CA inhibition by ZNS might contribute to its antiepileptic activity. Clear experimental evidence that probes this last point is still lacking; indeed, the anticonvulsant effect of ZNS is not usually attributed to its CA inhibition properties [10].

Topiramate (TPM), known with the brand name Topamax, is a widely used drug for epilepsy treatment. Its use was approved in 1996 as an adjunctive and monotherapy in children as well as in adults for partial-onset seizures and for drug-resistant patients with primary or secondary generalized tonic–clonic seizures. It is considered a broad-spectrum agent, also effective as a prevention therapy for migraine headaches. Moreover, TPM is highly bioavailable and presents low protein binding [9]. Its effectiveness was probed in several animal model of epilepsy: MES model in rodents [93], amygdala kindling, a model for complex partial seizures [94], genetic absence epilepsy in rats [95], kainic acid (KA) model of temporal lobe epilepsy [96] and in an animal model for Dravet Syndrome, a severe paediatric genetic epilepsy [97]. TPM is currently used as an adjunctive therapy in children with this last form of highly pharmaco-resistant epilepsy [98]. Despite its high efficiency, fairly long-term, TPM use in patients might lead to a quite consistent list of side effects: dizziness, nervousness, anxiety and depression, confusion, coordination abnormality, loss of appetite, sensory distortion and cognitive impairment, just to mention a few. An extended release formulation (TPM-XR) has been available since 2014. TPM-XR has shown both efficacy and tolerability, though still with some adverse effects when used as either a monotherapy or an adjunctive therapy in epilepsy patients with focal or generalized seizures [99].

Topiramate inhibits all CA isoforms, in particular it strongly inhibits cerebral CA II and CA VII [40]. CO_2_ retention [9], inhibition of the GABA-A mediated depolarization [100] and decrement of the initial alkalization accompanying abnormal neural activity [101], observed following TPM application, might be considered as consequences of the TPM-induced CA inhibition and might account for its anticonvulsant propriety. The anticonvulsant effect of TPM can also be due to the TPM capacity to alter neural excitability through other mechanisms, apparently unlinked with CA inhibition: TPM enhances inhibition by increasing the frequency of GABA-mediated chloride channel opening [102] by upregulating GABA levels [103] and by increasing potassium conductance [100], while it reduces neuronal excitation by inhibiting kainate-type glutamate receptors [104,105] and by blocking voltage-gated sodium channels [106] and L-type voltage-sensitive calcium channels [107].

Despite the existence of many studies devoted to analyzing the effects of these CAIs in the epileptic brain, the mechanisms by which they exert their antiseizure effects are not fully clarified, and new investigations are needed to elucidate them. Moreover, as reported above, it is clinically demonstrated that some of these CAIs do not show full effectiveness in long-term epilepsy therapy and that their use entails side effects, sometimes even disabling, in patients. Therefore, the development of new CA inhibitors with long lasting efficacy and without unpleasant side effects is required. For this reason, in the last years, researchers have spent a great deal of effort in the design of new generation CAIs that may have high specificity and a fully satisfactory clinical exploitability in epilepsy.

## 5. Anticonvulsant CAIs Design Strategy

Besides the clinically used CAIs, in the last years, researchers have spent a great deal of effort in the design of a new generation of CAIs that may have high specificity and a fully satisfactory clinical exploitability in epilepsy. This major area of research delves into the design of potent and isoform-selective CAIs to improve their tolerance and safety. One strategy to develop compounds displaying effective anticonvulsant activity is to target CA II and CA VII isoforms that are present in the brain. As mentioned before, CA II is overexpressed in numerous CNS disorders including epilepsy and CA VII is widely expressed in the most affected regions during the epileptic action. 

In the past decades, X-ray crystallographic studies revealed that the sulfonamide group (-SO_2_-NH_2_) binds in its deprotonated form, in the active site of CA, blocking catalysis efficiently [108]. This recognized zinc binding group (ZBG) was then incorporated in various scaffolds to develop excellent isoform-selective CA inhibitors [109]. 

**Figure 9:**
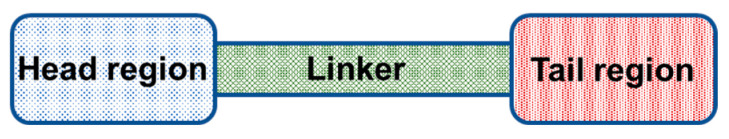
Graphical representation of CAIs pharmacophore.

Benzensulfonamide scaffold (Ar-SO_2_NH_2_) has emerged as a pharmacophoric head group to design potent and isoform-selective CAIs. The head region (ZBG), which coordinate with zinc ions in the active site of CA, is connected through a linker/spacers to various tail regions constituting the pharmacophore frame, Figure 9. Several benzensulfonamide (ZBG)-based inhibitors with excellent inhibitory activity against various CA isoforms, were synthetized by appending alkyl/aryl/hetero aryl/sugar scaffolds (tails) using several types of linkers such as carboxamide, urea, thiourea, acid hydrazide and amide.

Recently, several benzensulfonamide CAIs that had been shown to be selective in vitro towards CA II and CA VII, have given promising results also in in vivo animal models. The in vivo evaluation of the anticonvulsant activity was performed using the maximal electroshock (MES test) and subcutaneous pentylenetetrazol (PTZ) seizure test on mice; the two common accepted tests in antiepileptic drug discovery [42,110].

**Figure 10:**
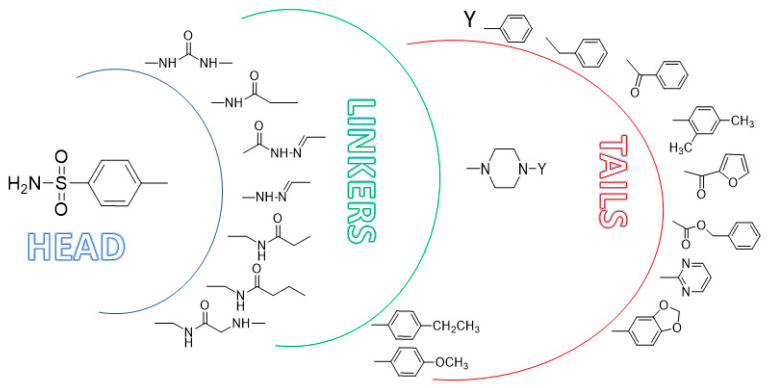
Graphical representation of molecular fragments presents in safe and orally active CAII and CAVII benzensulfonamide-based inhibitors displaying anticonvulsant activity on in vivo tests.

Figure 10 displays the pharmacophoric portions present in compounds that have shown the best anticonvulsant activity in in vivo animal models [42,111,112].

In detail, the head is characterized by benzensulfonamide portion, the linkers are of several types such as carboxamide, urea, acid hydrazide and amide, etc. Finally, the best tails consist of phenyl para substituted group or N-substituted piperazine moiety. The latter is functionalized on a nitrogen atom with various Y groups, as reported in Figure 10.

The flexibility of the linker is considered an important feature to allow the interaction of the inhibitors into the CA active site and provide selective inhibitors. The carboxamide linker and the more flexible acetamide/propionamide linkers connected to the N-substituted piperazine tail has produced effective and selective CA II and CA VII inhibitors along with anticonvulsant action, orally active and non-toxic in neuronal cell lines and in animal models.

## 6. Conclusions

Although some aspects remain to be addressed, most of the scientific evidence at our disposal agrees that CAs are involved in epileptic mechanisms. In particular, CA II, CA VII and CA XVI seem to play a major role in epilepsy, but their exact mode of action is still unclear. A clear understanding of this issue would open new perspectives for therapeutic intervention in epilepsy.

CAI antiepileptic proprieties have been well-known since the 1950s–1960s. In particular, ACZ, ZNS and TPM have been exploited for epilepsy treatment. ZNS and TPM are currently considered as seizure medicines widely used in clinical practice as adjunctive therapies. However, they inhibit a broad range of CA isoforms and their mechanism of action cannot be solely attributed to the inhibition of CAs. Moreover, their clinical use has shown unpleasant side effects in patients. To overcome these issues, in the last years, several molecules have been synthesized with the aim to inhibit specific CA isoforms and to exploit them as anticonvulsants with less side effects. This new generation of selective CAIs might not only improve our understanding of the functional role of the specific CA isoforms particularly critical for epilepsy, but might also represent new promising therapeutic options to be implemented, alone or in combination with other AEDs, in epilepsy treatment.
